# Gene Expression Profiles Underlying Selective T-Cell-Mediated Immunity Activity of a Chinese Medicine Granule on Mice Infected with Influenza Virus H1N1

**DOI:** 10.1155/2014/976364

**Published:** 2014-01-08

**Authors:** Na-na Lu, Qi Liu, Li-gang Gu, Shi-jie Ge, Jun Wu, Qiu Ze-ji, Ze-ji Qiu, Hong-chun Zhang, En-xiang Chao, Zhuo-nan Yu

**Affiliations:** ^1^Laboratory of Chinese Medicine on Viral Disease, Basic Medical College, Beijing University of Chinese Medicine, Beijing 100029, China; ^2^Shanxi University of Traditional Chinese Medicine, Taiyuan, Shanxi 030024, China; ^3^Department of Respiratory Medicine, China-Japan Friendship Hospital, Beijing 100029, China

## Abstract

A Chinese medicine granule, Shu-Feng-Xuan-Fei (SFXF), is critical for viral clearance in early phase of influenza virus infection. In this study, 72 ICR mice were randomly divided into six groups: normal control group, virus control group, Oseltamivir group, low-dose SFXF, medium-dose SFXF, and high-dose SFXF. Mice were anesthetized and inoculated with 4LD50 of influenza virus A (H1N1) except normal control group. Oseltamivir group received 11.375 mg*·*kg^−1^ *·*d^−1^ Oseltamivir Phosphate. SFXF 3.76, 1.88 and 0.94 g*·*kg^−1^ *·*d^−1^ were administrated to mice in all SFXF groups. Each group was in equal dose of 0.2ml daily for 4 consecutive days. Mice were sacrificed and then total RNA was extracted in lung tissue. Some genes involved in T-cell-mediated immunity were selected by DNA microarray. These candidate genes were verified by Real-Time PCR and western immunoblotting. Compared with virus control group, in Toll-like receptor signaling pathway, 12 virus-altered genes were significantly reduced following medium-dose SFXF treatment. Eighteen antigen processing presentation-associated genes were upregulated by medium-dose SFXF. In the process of T cell receptor signaling pathway, 19 genes were downregulated by medium-dose SFXF treatment. On exploration into effector T cells activation and cytokines, all of altered genes in virus control group were reversed by medium-dose SFXF. Real-time PCR and western immunoblotting showed that the regulation of medium-dose SFXF in IL-4, IFN-*γ*, TNF-*α*, IL-1*β*, TLR7, MyD88, p38, and JNK was superior to Oseltamivir and high-dose SFXF group. Therefore, SFXF granules could reduce influenza infected cells and activation of T cells.

## 1. Introduction

Influenza virus A (H1N1) has emerged every year and remained a public health threat worldwide. Influenza virus not only can damage the epithelial cells, of the lung and airways, but also may lead to complications of extrarespiratory diseases. Today the main option for prevention and treatment is the neuraminidase inhibitor (NAI), Oseltamivir Phosphate, which prolongs influenza virus shedding, decreases the signs of infection, reduces the spread of H1N1pdm influenza virus in the lungs of ferrets, and impedes the development of viral pneumonia [1]. However, the risk of Oseltamivir for virus strain resistance, possible side effects, and financial cost outweigh the small benefits for the prophylaxis and treatment of healthy individuals [2]. To address these issues, other effective alternative treatments of symptomatic influenza, especially Chinese herbal granules, will be additionally needed to have maximal reduction in incidence and mortality of influenza. For example, it was reported that Yin-Qiao-San could reduce time of fever resolution in patients with H1N1 influenza virus infection [3]. This paper studies Shu-Feng-Xuan-Fei granules (SFXF) whose major ingredients are based upon classical Yin-Qiao-San formula. Before this study, based on the guidelines issued by the Chinese Ministry of Health, the data of pyretic patients in fever clinic of Chinese-Japanese Friendship Hospital were collected by rapid influenza diagnostic tests (RIDT) from February to April, 2012. Clinical trials of this herbal granule had demonstrated its efficacy in reducing the duration of fever in patients with influenza A (H3N2) virus infection [4]. However, the molecular mechanisms of this herbal granule are still unclear. DNA microarray analysis has emerged as an important tool in the characterization of changes in host gene expression following infection by influenza virus. In this study, we reported a systematic evaluation of its immune-modulatory activities *in vivo*.

## 2. Methods and Materials

### 2.1. Virus

The virus strain used in the study was a mouse-adapted strain of influenza A/FM/1/47 (H1N1), kindly offered by the Chinese Center for Disease and Prevention. Virus was grown in 9-day-old embryonated chicken eggs and virus-containing allantoic fluids were used in experiments. The supernatants were clarified, harvested, and stored at −70°C. The viral titer in the viral stocks, or tissue homogenates, was determined using a medium lethal dose (LD50) assay and then calculated by the Muench-Reed method (1938) [5]. The medium lethal dose (LD50) was determined in mice after serial dilution of the stock. Two times value of LD50 were used for viral challenge in all of the experiments. LD50 value = 10^−2.24^.

### 2.2. Preparation of Herbal Extracts

Oseltamivir Phosphate was purchased from the F. Hoffmann-La Roche Ltd. (Basel, Swiss) (no. J20040058). SFXF granule was manufactured and provided by Beijing Tcmages Pharmaceutical Co., Ltd. SFXF consists of *lonicera japonica* (10 g), *forsythia* (10 g), *dyers woad leaf* (10 g), *great burdock achene* (10 g), *periostracum cicadae* (8 g), *thunberg fritillary bulb* (10 g), *scutellaria* (10 g), *radix asteris* (15 g), almond (10 g), *platycodon grandiflorum* (10 g), *glycyrrhiza uralensis* (6 g), and *radix isatidis* (10 g). One hundred grams of granules was dissolved in 500 mL water and kept at 4°C over night. The autoclaved herbal juice was then concentrated by continuous freeze-drying operation for 72 hours until the solvent was completely removed. These granules were kept in airtight containers at −70°C until further use.

### 2.3. Animal Experiments

Seventy-two male ICR mice (13 to 15 g body weight) were purchased from SPF Lab Animal, Ltd. (Beijing, China). All mice were housed at an animal facility under specific-pathogen-free conditions. Mice were housed in individually ventilated cages provisioned with water and standard feed and were monitored daily for health and condition. All H1N1 *in vivo *experiments were performed under biosafety level 3 enhanced (BSL3+) containment. Mice were exposed to 4LD50 of virus by the intranasal (i.n.) route. All animal experiments were handled to the protocol approved by the university animal committee. According to random number table, 72 ICR mice were randomly divided into six groups (*n* = 12): normal control group (N), virus control group (M), Oseltamivir group, low-dose SFXF (SL), medium-dose SFXF (SM), and high-dose SFXF (SH). Mice were anesthetized with 2,2,2-tribromoethanol in tert-amyl alcohol and inoculated (i.n.) with 4LD50 of virus except normal control group. Normal control group was given isotonic saline 0.05 mL in nasal drops. After 2 hours of inoculation, Oseltamivir group received 11.375 mg·kg^−1^·d^−1^ Oseltamivir Phosphate. SFXF 3.76, 1.88, and 0.94 g·kg^−1^·d^−1^ were administrated to mice in SL, SM, and SH groups by gastric irrigation, respectively. The medium dosage SFXF granule for mouse study was equivalent to the human dosage in clinical practice, while the SL was half and the SH was twice of the human clinical dosage, respectively. Each group was in equal dose of 0.2 mL daily for 4 consecutive days. Total RNA was extracted in each group.

### 2.4. Microarray Data Analysis

One microgram of total RNA was prepared for the cDNA reversed transcription reaction and performed using Amino Allyl MessageAmp II aRNA Amplification Kit (Ambion no. AM1753, CA, USA) according to manufacture's instructional resources information system. Double stranded cDNA was synthesized and as a template followed by an *in vitro* transcription reaction to amplify aRNA while biotin was incorporated into the synthesized aRNA probe. 40 *μ*g of unlabeled aRNA was subsequently used in each labeling reaction to generate sufficient Cy5-labeled aRNA for the microarray hybridization in triplicate. Before the microarray hybridization, the Cy5-labeled aRNAs were fragmented by using the reagents and protocol provided in Ambion RNA Fragmentation Reagents kit (Ambion Inc., Austin, TX). Each 10 *μ*g fragmented Cy5-labeled aRNA was suspended in OneArray hybridization buffer at a final volume of 180 *μ*L for microarray hybridization to the mouse Whole Genome One Array Version 5.1 (HOA 5.1, Phalanx Biotech Group, Inc., Taiwan). After an overnight hybridization at 50°C, nonspecific binding targets were washed out three times (Wash, 42°C, 5 min; Wash II, 42°C, 5 min; Wash III 25°C, 5 min). The arrays were dried by centrifugation and were scanned by AXON4000B scanner (Molecular Devices, CA, USA). The fluorescent intensities of each spot were analyzed by GenePix 4 (Molecular Device, CA, USA). And then the data were averaged from the three technical replicates and were normalized using Rosetta Resolver System software (Rosetta Biosoftware, USA). The spots that flags reported as −50 were filtered out. Rosetta error models were available in the Rosetta Resolver system for gene expression analysis in two different groups. Five pairwise comparisons were performed. For example, the comparison between normal control group and virus control group was scattered, indicating that a large number of genes were altered in response to H1N1-infected mouse lung. Changes in gene expression were attributable to the SH, SM, SL, and Oseltamivir intervention which were done by comparing genes in virus control group. Gene Cluster 3.0 and Eisen's Treeview software (Stanford University) were used to compare similarities among individual samples. The log2 transformed intensity of any two gene expression profiles was plotted and compared, while a fold change (FC) over 2 or under 0.5 (|log_2_FC| ≥ 1), as well as *P* value <0.05, was considered to indicate differential expression. Genes whose relative expression levels showed log_2_FC ≥ 1 and *P* < 0.05 were considered significantly upregulated, and those with log_2_FC ≤ −1 and *P* < 0.05 were considered significantly downregulated. The correlation of expression profiles between biological replicates and treatment conditions was demonstrated by unsupervised hierarchical clustering analysis. The functions of differentially expressed genes involved in immunomodulatory biological pathways were analyzed by Kyoto Encyclopedia of Genes and Genomes (KEGG) Pathway databases in Database for Annotation, Visualization, and Integrated Discovery (DAVID, http://david.abcc.ncifcrf.gov/).

### 2.5. Real-Time PCR Analysis

Real-Time PCR, a technology used for the detection and quantification of RNA targets, is considered as “gold standard” for verifying the microarray data. Total RNA was extracted from 50 to 100 mg of lung tissue with TRIzol (Invitrogen) according to the protocol described for the SYBR Green PCR kit (Takara Bio Inc., Shiga, Japan). For smaller samples, homogenization in liquid nitrogen could be done using mortar and pestle. Phase separation was achieved by adding chloroform (0.2 mL/mL Trizol), vortexing, and incubation at room temperature for 3 min. The tubes were centrifuged at 12000 ×g at 4°C for 15 min. The top, aqueous phase was transferred into a fresh RNA tube. Isopropanol was added and samples were mixed thoroughly and incubated at room temperature to precipitate RNA. Isopropanol was then replaced by 75% ethanol (1 mL/mL Trizol), mixed thoroughly, and centrifuged at 7500 ×g for 5 min at 4°C. The supernatant was removed. The RNA pellet redissolved in DEPC-H_2_O (50 *μ*L). The primer sequences of the expected PCR products were as follows: GAPDH (141 bp), Forward primer: 5′-GCAAGTTCAACGGCACAG-3′, and reverse primer: 5′-CGCCAGTAGACTCCACGAC-3′; IL-4 (142 bp), forward primer: 5′-TGTACCAGGAGCCATATCCA-3′, and reverse primer: 5′-CTGTGGTGTTCTTCGTTGCT-3′; IFN-*γ* (179 bp), forward primer: 5′-AGGCCATCAGCAACAACATA-3′, and reverse primer: 5′-TGAGCTCATTGAATGCTTGG-3′; TNF-*α* (133 bp), forward primer: 5′-CCAAAGGGATGAGAAGTTCC-3′, and reverse primer: 5′-CTCCACTTGGTGGTTTGCTA-3′; IL-1*β* (130 bp), forward primer: 5′-TCAGGCAGGCAGTATCACTC-3′, and reverse primer: 5′-AGGATGGGCTCTTCTTCAA-3′; IL-8 (242 bp), forward primer: 5′-CTCTTGGCAGCCTTCCTGAT-3′, and reverse primer: 5′-ACAACCCTCTGCACCCAGTT-3′; ICAM-1 (122 bp), forward primer: 5′-CCTCCGGACTTTCGATCTT-3′, and reverse primer: 5′-GAGCTTCAGAGGCAGGAAAC-3′; TLR7 (117 bp), forward primer: 5′-ACGCTTTCTTTGCAACTGTG-3′, and reverse primer: 5′-TTTGTGTGCTCCTGGACCTA-3′; MyD88 (136 bp), forward primer: 5′-TGGTGGTTGTTTCTGACGAT-3′, and reverse primer: 5′-GGAAAGTCCTTCTTCATCGC-3′; JNK (128 bp), forward primer: 5′-ATGCAAATCTTTGCCAAGTG-3′, and reverse primer: 5′-AGGCTTTAAGTCCCGATGAA-3′; p38 (195 bp), forward primer: 5′-AAGCCATGAGGCAAGAAACT-3′, and reverse primer: 5′-TCATCAGGGTCGTGGTACTG-3′. Preincubation was performed at 94°C for 1 min, followed by amplification in 40 cycles at 94°C for 8 s, 60°C for 34 s, and finally, during slow heating up, 72°C for 10 min. Fold changes of expressions relative to vehicle controls were determined after normalization to glyceraldehydes-3-phosphate dehydrogenase (GAPDH) gene. Relative quantification is generally calculated with the 2^−ΔΔCT^ formula by the comparative CT method [6]. The ΔCT value is determined by subtracting the average GAPDH CT value from the average target gene CT value (ΔCT = CT target gene − CT GADPH). The calculation of ΔΔCT involves subtraction by the ΔΔCT calibrator value (ΔΔCT = ΔCT each other sample − ΔCT normal control sample). The fold change for each of treated and virus control samples is relative to the normal control sample = 2^−ΔΔCT^.

### 2.6. Western Immunoblotting

All selected RNA samples were the same as the ones from those used in the DNA microarray assay and real-time PCR assay. For smaller samples, homogenization in liquid nitrogen could be done using mortar and pestle. The tissue was placed to the precooling Eppendorf tube and we added 50 *μ*L lysis buffer rapidly in the tube. Then the tube was placed on ice for 20 min and centrifuged at 16,000 ×g at 4°C for 20 min. We transferred the supernatant to a fresh tube kept on ice and discarded the pellet. We added an equal volume of 2 × SDS Sample Buffer and boiled each cell lysate in sample buffer at 100°C for 5 minutes. Protein lysates were denatured, subjected to 4 to 15% SDS-polyacrylamide electrophoresis gradient gels (Bio-Rad), and transferred to nitrocellulose membranes. The samples were then electrotransferred onto the nitrocellulose membrane. Membranes were incubated overnight and probed with rabbit anti-mouse IL-4 (bs0581R) and IFN-*γ* (bs0480R) polyclonal antibody (Biosynthesis Biotechnology Co., LTD, Beijing), rabbit anti-mouse TNF-*α* (BC0088) and IL-1*β* (BA2782) polyclonal antibody (Boster Biological Co., LTD, WuHan), rabbit anti-human JNK (4764S) and p38 (9252S) polyclonal antibody (Cell Signaling Technology, Inc), rabbit anti-mouse MyD88 (bs1047R) monoclonal antibody (Biosynthesis Biotechnology Co., LTD, Beijing), rabbit anti-human TLR7 (bs6601R) polyclonal antibody (Biosynthesis Biotechnology Co., LTD, Beijing), and rabbit anti-GAPDH (Sigma, USA). Membranes were then washed with TTBS four times for 5 min each and incubated with 1 : 2000 dilution of HRP-labeled Goat Anti-Rabbit IgG (H + L) (Zhongshan Golden Bridge Bio-technology, Beijing) for 1 h at room temperature. Immunoreactive bands were detected using ECL reagents (Santa cruz Biotechnology, Inc., USA) and IPP software. Next we also calculated a relative intensity, using our standard as the common point of comparison. Divide the absolute intensity of each sample band by the absolute intensity of your standard (GAPDH) to come up with a relative intensity for each sample band.

### 2.7. Statistical Analysis

The relative mRNA expression of the target gene was compared between all groups and analyzed by one-way analysis of variance (ANOVA). The differences between two groups were analyzed by Student's *t*-test, followed by a SPSS Statistics 17.0 Software. A *P* value of less than 0.05 (*P* < 0.05) was considered to be statistically significant. Results are presented as mean ± standard deviation (SD). Similar results were obtained in at least three independent experiments.

## 3. Result

### 3.1. Total Differentially Expressed Genes Induced by H1N1 Virus and Treatment

**Figure 1:**
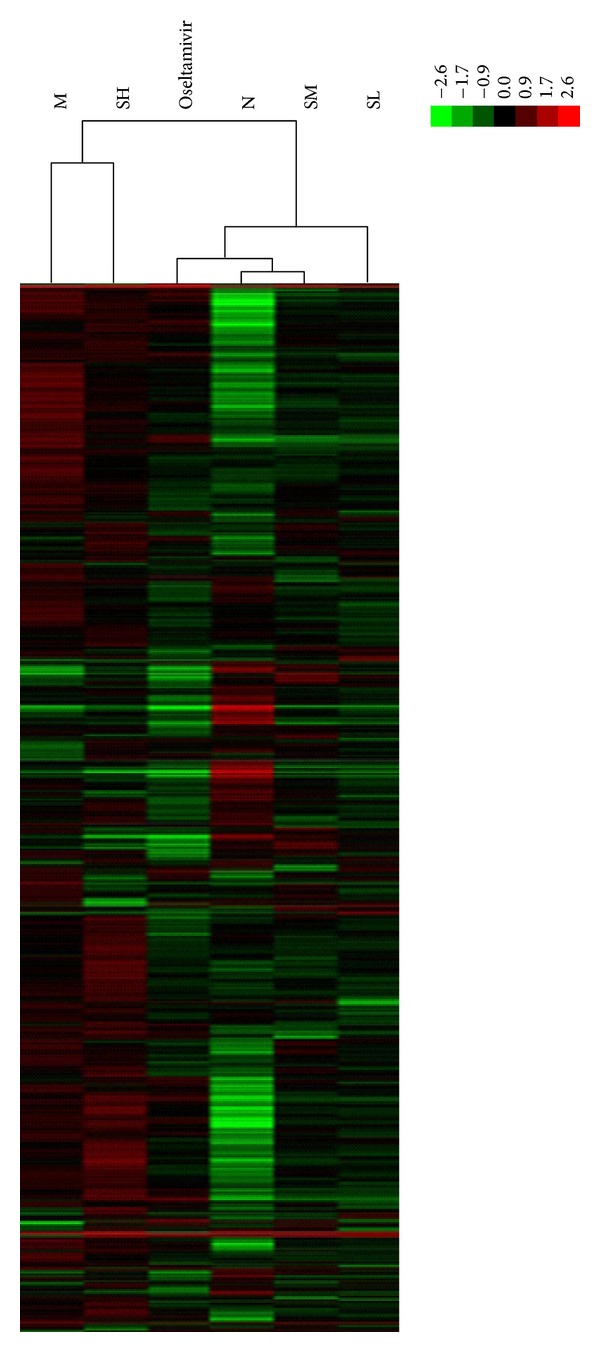
Two-way hierarchical clustering of selected genes in T cell recognition, activation, and proliferation activities was performed to visualize the correlations among the replicates and varying sample conditions. Up- and downregulated genes are represented in red and green colors, respectively. From left to right are virus control group, high-dose SFXF group, Oseltamivir group, normal control group, medium-dose SFXF group, and low-dose SFXF group.

The mouse whole genome microarray was used to perform a systematic alternation of the mRNA expression profiles after H1N1 infection and treatment. Hierarchical cluster analysis (HCA) was first conducted within ArrayTrack. A differentially expressed gene was identified following the criteria of a fold-change than 1 (up or down) and a *P* value less than 0.05 in comparison to the virus control group. Based on these two criteria, we identified virus control group upregulated 2670 genes expression and downregulated 2968 genes expression, compared to normal control group. Among these up-regulations of genes, 2358, 1877, 2659, and 1868 genes were significantly downregulated in response to the Oseltamivir group, the SH group, the SM group, and the SL group, respectively. Based on these downregulated genes in virus control group, up-regulations of 1849, 1302, 2216, and 1570 genes were identified in the Oseltamivir group, the SH group, the SM group, and the SL group, respectively, as shown in Figure 1.

### 3.2. Pathway Analysis of the Differentially Expressed Genes

In order to determine the correlation between the affected gene expression and the top 10 significant biological pathways induced by H1N1, the genes were grouped into functional categories and pathways based on the Kyoto Encyclopedia of Genes and Genomes (KEGG) database in Table 1. According to KEGG description, the results showed that the main pathways modulated T cell-mediated immunity during H1N1 infection, including Toll-like receptor signaling pathway, antigen processing and presentation, T cell receptor signaling pathway, and effector T cells activation and cytokines involved in T cell mediated immunity.

#### 3.2.1. Exploration into Toll-Like Receptor Signaling Pathway

**Figure 2:**
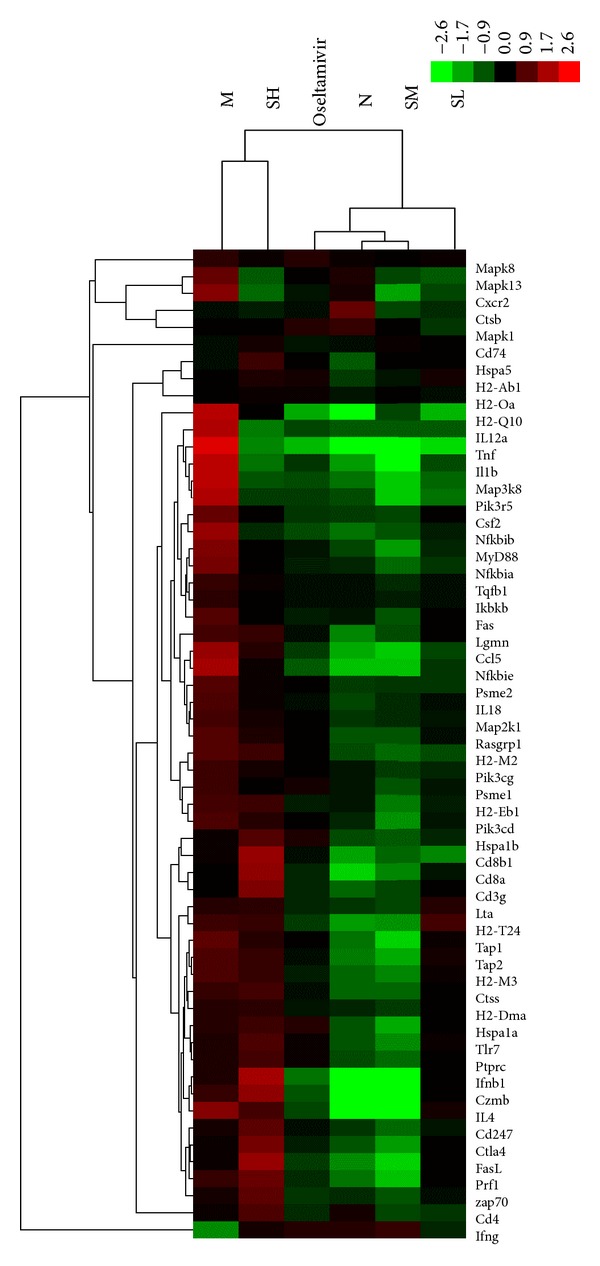
Two-way hierarchical clustering of selected genes in T cell recognition, activation, and proliferation activities was performed to visualize the correlations among the replicates and varying sample conditions. Up- and downregulated genes are represented in red and green colors, respectively. From left to right are virus control group, high-dose SFXF group, Oseltamivir group, normal control group, medium-dose SFXF group, and low-dose SFXF group.

Some key Toll-like receptor signaling pathway-related target genes were identified in the present study. A comprehensive examination of gene expression form lung tissues of normal and H1N1-infected mice showed 12 upregulated genes to be differentially expressed in the infective mice, such as *Tlr7*, *MyD88*, *Mapk8*, *Mapk13*, *Tnf*, *Il1b*, *Cxcr2*, *Ccl5*,* Csf2*, *Tgfb1*, *Il18,* and *Il12a*. All of these virus-altered genes were significantly reversed following the SM treatment. The prominent genes in these significantly changed networks were* Tnf *and* Il1b, *exhibiting 5.03- and 4.27-fold decreased expression exposed to the SM group, respectively. Except *Tlr7*, the other 11 genes above (i.e., *MyD88*, *Mapk8*, *Mapk13*, *Tnf*, *Il1b*,* Csf2*, *Cxcr2*, *Ccl5*, *Tgfb1*, *Il18*, and *Il12a*) were downregulated in response to Oseltamivir group. The SL and SH only could down-regulate 9 genes (*MyD88*, *Mapk8*, *Mapk13*, *Tnf*, *Il1b*, *Csf2*, *Cxcr2*, *Ccl5*, and *Il12a*) and 8 genes (*MyD88*, *Mapk8*, *Mapk13*, *Tnf*, *Il1b*, *Csf2*, *Cxcr2*, and *Il12a*), respectively, as shown in Figure 2 and Table 2.

#### 3.2.2. Exploration into Antigen Processing and Presentation

Some key antigen processing and presentation pathway-related target genes were identified in the present study. Twenty-three genes were identified as differentially expressed genes between virus-infected and normal mice, such as *Tap1*,* Tap2*,* Cd74*,* Ctsb*,* Ctss*,* Hspa5*,* Hspa1a*,* Hspa1b*,* H2-M2*,* H2-M3*,* H2-Oa*,* H2-Q10*,* H2-Dma*,* H2-Eb1*,* H2-Ab1*,* Lgmn*,* Lta*,* Psme1*,* H2-T24*,* Cd8a*,* Cd4*,* Cd8b1*, and* Psme2*. Compared with the virus control group, the gene expression result of the SM-treated mice was that a total of 18 antigen processing presentation-associated genes were differentially downregulated, among which 13 genes (*Tap1*,* Tap2*,* Hspa1a*,* Hspa1b*,* H2-M2*,* H2-M3*,* H2-Q10*,* Lta*,* Psme1*,* Cd8a*,* Cd8b1*,* H2-T24*, and* Psme2*) and 5 genes (*Ctss*,* H2-Dma*,* Lgmn*,* H2-Eb1*, and *Cd4*) belong to MHC-I and MHC-II family, respectively. In comparison with the virus control group, the result of the SL group was that a total of 5 antigen processing presentation-associated genes were differentially expressed, among which 4 genes (*H2-M2*,* H2-Q1*,* Cd8b1*, and* Psme2*) and 1 gene (*H2-Eb1*) belong to MHC-I and MHC-II family, respectively. Compared with the virus control group, in the SH group, *H2-Q10* and *Psme2 *belonging to MHC-I were differentially expressed in antigen processing presentation-associated genes as shown in Figure 2 and Table 3.

#### 3.2.3. Exploration into Process of T Cell Receptor Signaling Pathway

Some key T cell receptor signaling pathway-related target genes were identified in the present study. Sixteen differential genes were affected by H1N1 infection through comparison with the normal control group, such as* Cd3g*,* Cd247*,* Ptprc*,* Rasgrp1*,* Ctla4*,* Ikbkb*,* Mapk1*,* Map3k8*,* Nfkbie*,* Nfkbib*,* Nfkbia*,* Zap70*,* Pik3r5*,* Pik3cg*,* Map2k, *and* Pik3cd*. Fourteen genes (including *Ctla4*,* Cd247*,* Ptprc*,* Rasgrp1*,* Mapk1*,* Map3k8*,* Nfkbie*,* Nfkbib*,* Nfkbia*,* Zap70*,* Pik3r5*,* Pik3cg*,* Map2k1, *and* Pik3cd*) were downregulated by the SM treatment, of which 13 genes (*Ctla4*,* Cd247*,* Rasgrp1*,* Mapk1*,* Ikbkb*,* Map3k8*,* Nfkbie*,* Nfkbib*,* Nfkbia*,* Zap70*,* Pik3r5*,* Pik3cg, *and* Map2k1*) were also downregulated by the Oseltamivir group. The SL and SH group only could down-regulate 7 genes (*Rasgrp1*,* Map3k8*,* Nfkbie*,* Nfkbib*,* Nfkbia*,* Pik3r5,* and* Pik3cg*) and 4 genes (*Map3k8*,* Nfkbie*,* Nfkbib, *and* Pik3r5*), respectively, as shown in Figure 2 and Table 4.

#### 3.2.4. Exploration into Effector T Cells Activation and Cytokines Involved in T Cell Mediated Immunity

Some key effector T cells activation and cytokines-related target genes were identified in the present study. Influenza virus led to significant changes in the expression of 6 genes in virus control group, of which 5 genes (*Prf1*,* Gzmb*,* Fas*,* Fasl, *and* Il4*) were upregulated and 1 gene (*Ifng*) was downregulated in comparison with the normal control group. All of altered genes above were reversed by the SM group and Oseltamivir group. The notable genes in these significantly changed networks were* Gzmb *and* Il4, *exhibiting 4.79- and 5.19-fold decreased expression exposed to the treatment of the SM group, respectively. *Fasl*,* Fas*,* Il4, *and* Ifng* were found to be significantly altered by the SH group. Also down-regulation of *Il4, *as well as up-regulation of* Ifng*, was observed in the SL group as shown in Figure 2 and Table 5.

### 3.3. Confirmation of Microarray Data by Real-Time PCR

**Figure 3:**
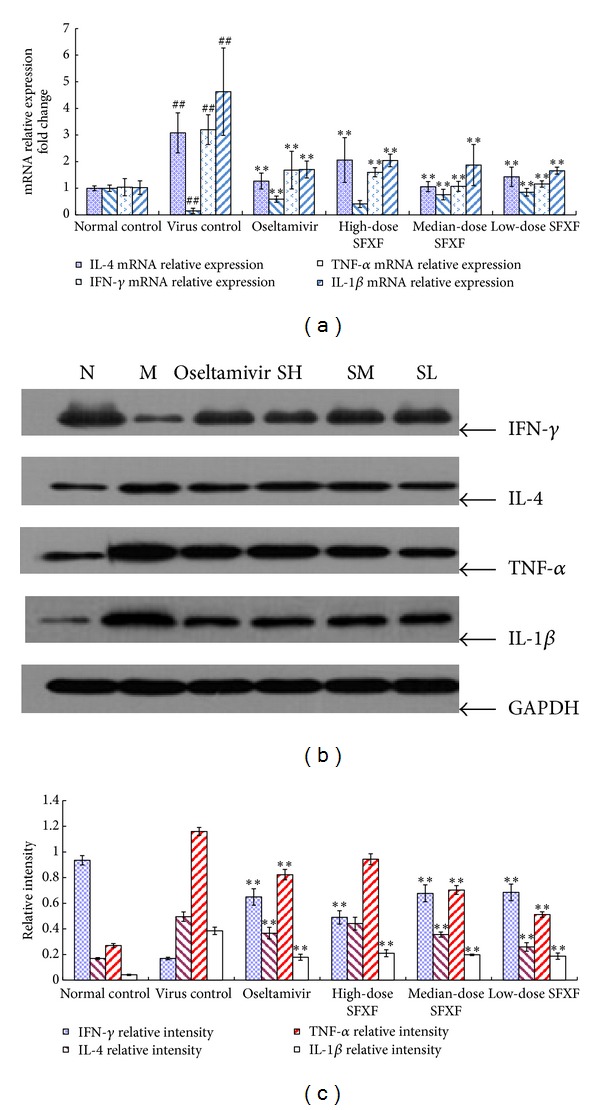
Mice were anesthetized and then infected intranasally by dropping 0.05 mL of influenza virus suspension (4LD_50_) except normal control group. High-dose SFXF (3.76 mg/kg), medium-dose SFXF (1.88 mg/kg), low-dose SFXF (0.94 mg/kg), or Oseltamivir (11.375 mg/kg) was administrated daily starting at 2 hours before the first viral infection until 4 days post-infection (4 dpi). Then total RNA was isolated from lung tissues of normal control, virus control, and Oseltamivir- and SFXF-treated mice and was analyzed by real-time PCR and western immunoblotting. (a) Relative quantification of the IL-4, IFN-*γ*, TNF-*α*, and IL-1*β* mRNA expressed in the mice lung tissue of six groups, with 12 mice in each group. The quantity of mouse IL-4, IFN-*γ*, TNF-*α*, and IL-1*β* mRNA expression was normalized to the mRNA expression of mouse GAPDH (a housekeeping gene). The quantities are shown as mean ± standard deviation (SD) and two indicated groups as determined by the Newman-Keuls multiple comparison test following one-way ANOVA. Compared with the normal control group: ^##^ *P* < 0.01 and compared with the virus control group: *P < 0.05, **P < 0.01. (b) Western immunoblottings of IL-4, IFN-*γ*, TNF-*α*, and IL-1*β* protein expressions in the mice lung tissue of six groups, with 12 mice in each group. In normal control group, mice were blank and not infected with H1N1 virus (N). Mice were infected with H1N1 virus but not treated with any drugs (M). And mice were infected with H1N1 virus with treatment of high-dose SFXF (SH), medium-dose SFXF (SM), low-dose SFXF (SL), and Oseltamivir. The total gray value of each band was determined using ECL reagents and IPP software. (c) Western immunoblottings of relative quantification of IL-4, IFN-*γ*, TNF-*α*, and IL-1*β* protein levels. The relative intensity data were shown as the ratios of the target gene intensity to GAPDH intensity. Data shown were the mean ± SD of three independent experiments. *P < 0.05 and **P < 0.01 versus the virus control group (virus only).

**Figure 4:**
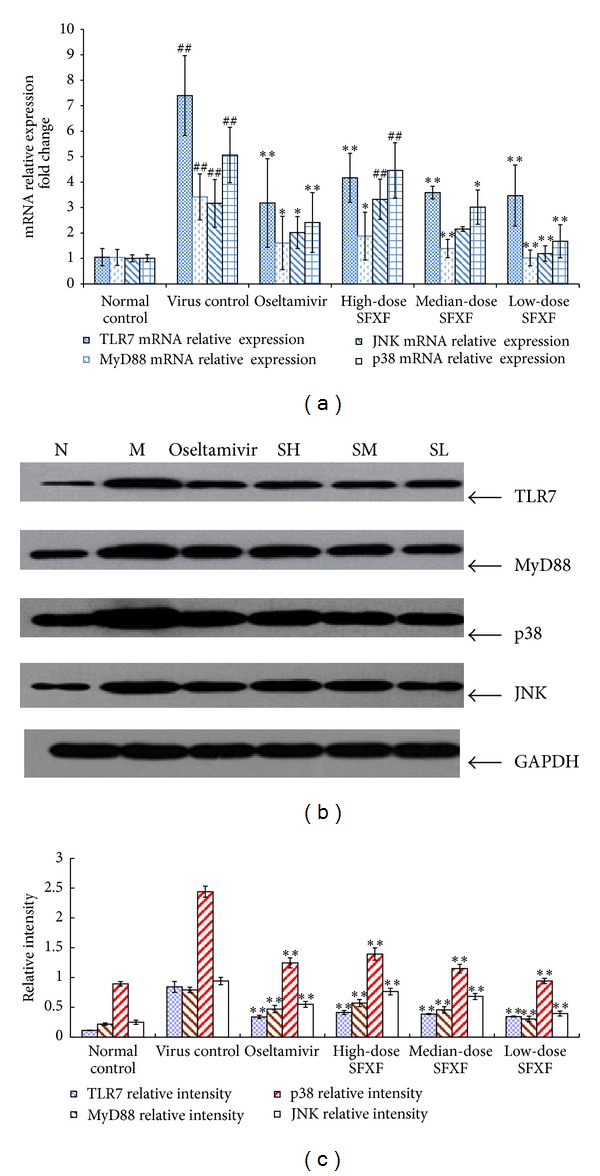
Mice were anesthetized and then infected intranasally by dropping 0.05 mL of influenza virus suspension (4LD_50_) except normal control group. High-dose SFXF (3.76 mg/kg), medium-dose SFXF (1.88 mg/kg), low-dose SFXF (0.94 mg/kg), or Oseltamivir (11.375 mg/kg) was administrated daily starting at 2 hours before the first viral infection until 4 days post-infection (4 dpi). Then total RNA was isolated from lung tissues of normal control, virus control, and Oseltamivir- and SFXF-treated mice and was analyzed by real-time PCR and western immunoblotting. (a) Relative quantification of the TLR7, MyD88, JNK, and p38 mRNA expressed in the mice lung tissue of six groups, with 12 mice in each group. The quantity of mouse TLR7, MyD88, JNK, and p38 mRNA expression was normalized to the mRNA expression of mouse GAPDH (a housekeeping gene). The quantities are shown as mean ± standard deviation (SD) and two indicated groups as determined by the Newman-Keuls multiple comparison test following one-way ANOVA. Compared with the normal control group: ^##^ *P* < 0.01 and compared with the virus control group: *P < 0.05, **P < 0.01. (b) Western immunoblottings of TLR7, MyD88, JNK, and p38 protein expressions in the mice lung tissue of six groups, with 12 mice in each group. In normal control group, mice were blank and not infected with H1N1 virus (N). Mice were infected with H1N1 virus but not treated with any drugs (M). And mice were infected with H1N1 virus with treatment of high-dose SFXF (SH), medium-dose SFXF (SM), low-dose SFXF (SL), and Oseltamivir. The total gray value of each band was determined using ECL reagents and IPP software. (c) Western immunoblottings of relative quantification of TLR7, MyD88, JNK, and p38 protein levels. The relative intensity data were shown as the ratios of the target gene intensity to GAPDH intensity. Data shown were the mean ± SD of three independent experiments. *P < 0.05 and **P < 0.01 versus the virus control group (virus only).

**Figure 5:**
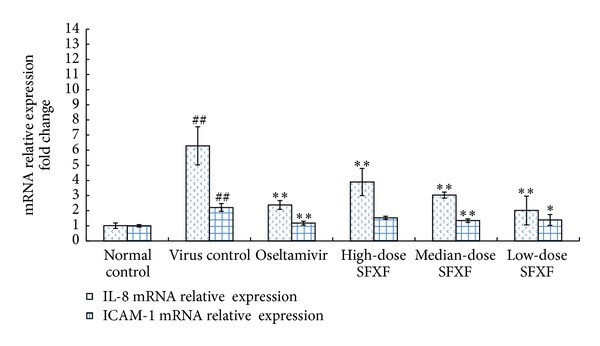
Mice were anesthetized and then infected intranasally by dropping 0.05 mL of influenza virus suspension (4LD_50_) except normal control group. High-dose SFXF (3.76 mg/kg), medium-dose SFXF (1.88 mg/kg), low-dose SFXF (0.94 mg/kg), or Oseltamivir (11.375 mg/kg) was administrated daily starting at 2 hours before the first viral infection until 4 days postinfection (4 dpi). Then total RNA was isolated from lung tissues of normal control, virus control, and Oseltamivir- and SFXF-treated mice and was analyzed by real-time PCR. Relative quantification of the IL-8 and RANTES mRNA had expressions in the mice lung tissue of six groups, with 12 mice in each group. The quantity of mouse IL-8 and RANTES mRNA expression was normalized to the mRNA expression of mouse GAPDH (a housekeeping gene). The quantities are shown as mean ± standard deviation (SD) and two indicated groups as determined by the Newman-Keuls multiple comparison test following one-way ANOVA. Compared with the normal control group: ^##^ *P* < 0.01 and compared with the virus control group: *P < 0.05, **P < 0.01.

Real-time PCR was adopted to validate some selected genes whose expression changes were seen using microarrays, including the up-regulation of IL-1*β*, TNF-*α*, IL-4, TLR7, MyD88, JNK, p38, IL-8, and RANTES gene expressions and the down-regulation of IFN-*γ* gene expression. All selected RNA samples were the same as the ones from those used in the DNA microarray assay. Compared with normal control group, in virus control group, the expression of IFN-*γ* mRNA was significantly downregulated, while the expression of IL-1*β*, TNF-*α*, IL-4, TLR7, MyD88, JNK, p38, IL-8, and RANTES mRNA was markedly upregulated (all *P* < 0.05). Compared with virus control group, in all the treatment groups except the SH group, the expressions of IFN-*γ* mRNA were significantly upregulated, while IL-1*β*, TNF-*α*, IL-4, TLR7, MyD88, JNK, p38, IL-8, and RANTES mRNA were markedly downregulated. These differential genes in virus control group were the most significantly altered by the medium SFXF treatment. The regulation of the SM group in IL-1*β*, TNF-*α*, IL-4, TLR7, MyD88, JNK, p38, IL-8, and RANTES was superior to Oseltamivir, the high- and low-dose SFXF. As expected, real-time PCR data were consistent with the results of microarray assay as shown in Figures 3, 4, and 5.

### 3.4. Determination of IL-4, IFN-*γ*, IL-1*β*, TNF-*α*, TLR7, MyD88, JNK, and p38 Proteins in Lung Tissues by Western Immunoblotting

The levels of IL-1*β*, TNF-*α*, IL-4, TLR7, MyD88, JNK, and p38 were low and IFN-*γ* was high in the lung tissue of the normal control group. The protein levels of IL-1*β*, TNF-*α*, IL-4, TLR7, MyD88, JNK, and p38 in the H1N1 infection group were higher than those in normal control group (*P* < 0.05). In infected mice, the protein level of IFN-*γ* was lower than that in uninfected mice. The majority of upregulated IL-1*β*, TNF-*α*, IL-4, TLR7, MyD88, JNK, and p38 were significantly suppressed, while the level of IFN-*γ* was also converted to increase with treatment of SFXF in all doses and Oseltamivir group, which meant that significant difference was found between the virus control group and all treatment groups. After the treatment of the SM, all variants had turned to baseline levels, which showed no statistical difference from Oseltamivir group as shown in Figures 3 and 4.

## 4. Discussion

Chinese compound medicine has become one of the most popular choices on therapeutic treatment of influenza in the Orient, because it has such advantages as multiple pathways, multitargets, and low side effect, for example, Xiao-qing-long-tang [3]. Unfortunately, the interaction of complicated composition, the important signaling pathways, and the efficacy of Chinese traditional medicine at the transcriptional level all challenge our biotechnique to identify precise molecular therapeutic targets. Therefore, the whole-genome DNA microarray has been proved to be an unbiased and high-throughput approach to thoroughly analyze virus infection and antiviral treatment by monitoring gene changes in deletion variants.

In this study, we studied the gene transcription profiles of the lung tissue of mice infected with H1N1 virus for 4 consecutive days. The major pathways involved in T-cell mediated immunity in response to H1N1 infection were mostly upregulated, such as Toll-like receptor signaling pathway, antigen processing and presentation, and T-cell receptor signaling pathway, as well as effector T cells activation and cytokines in cell-mediated immunity.

Since the largest number of differentially expressed genes was detected in the medium-dose SFXF treatment, we focused further analyses on gene functions and pathways primarily for the medium-dose SFXF treatment. In terms of Toll-like receptor signaling pathway, TLR7 (*Tlr7*) senses single-stranded RNA from influenza virus within the endosomes and induces the downstream signaling pathway involving MyD88 (*Myd88*)/IRAK/TRAF6, which activated the p38 (*Mapk13*) and JNK (*Mapk8*), causing proinflammatory cytokines (i.e., TNF-*α*, IL-1*β*, IL-8, IL-12, IL-18, GM-CSF, and RANTES) production to control and potentially eradicate virus infections [7]. In T cells, TLR7 activation mediates inflammation, cross presentation of cell-associated antigens, and cross priming of CD8+ T cells upon lung IVA infection [8]. MyD88 is crucial for Th1 cell responses against primary IVA infection [9]. JNK is required for polarized differentiation of T-helper cells into Th1 cells. TCR-activated p38*α* and p38*β* are important and redundant positive regulators of T-cell proliferation and Th skewing [10]. Once infected with influenza virus, proinflammatory cytokines are released from airway epithelial cells to trigger inflammatory responses, as the body's first line of defense against infection or injury. Otherwise, overexpression of cytokine storms in the host can cause significant pathology and ultimately death. In the present study, overexpression of cytokines, including TNF-*α* (*Tnf*), IL-1*β* (*Il1b*), IL-8 (*Cxcr2*), and RANTES (*Ccl5*), led to inflammation-induced tissue pathology in virus control group. SFXF in medium-dose significantly decreased the TLR7, MyD88, p38, JNK, TNF-*α*, IL-1*β*, IL-8, and RANTES mRNA expression (*P* < 0.05 or *P* < 0.01), compared with the virus control group. The medium-dose SFXF may provide evidence that pathway-specific in Toll-like inhibition is possible. SFXF formula may prevent the overexuberant activation of cytokines, contribute to the resolution of inflammation, and inhibit inflammatory immunopathogenesis from influenza-induced injury.

Following infection with H1N1, viral proteins were synthesized in the infected cell for the virus control group. The significantly changed genes after the medium-dose SFXF administration were that a total of 13 genes (*Tap1*,* Tap2*,* Hspa1a*,* Hspa1b*,* H2-M2*,* H2-M3*,* H2-Q10*,* Lta*,* Psme1*, *H2-T24*,* Psme2*, *Cd8a, *and* Cd8b1*) were belonging to pathway of MHC-I antigen processing and presentation. The processing of antigens is regulated by two distinct pathways, one requiring PA28 and the other hsp70 [11]. Heterodimer of PA28a (*Psme1*) and PA28b (*Psme2*) induced by membrane Lta (*Lta*) results in proteolysis of intracellular proteins to generate class I binding peptides. Hsp70 (*Hspa1a*,* Hspa1b*) is an effective molecular stimulator in the induction of CD8 T cells to inhibit influenza virus ribonucleoprotein (RNP) complex [12]. Then Tap1 (*Tap1*) and Tap2 (*Tap2*), as transporters of antigenic peptides, together select and pump cytosolic peptides into the lumen of endoplasmic reticulum, where major histocompatibility complex I (MHC- I) genes (*H2-M2*,* H2-M3*,* H2-Q10,* and *H2-T24*) were upregulated. Binding of peptide stabilizes the class I molecule and facilitates its transport to the cell surface. CD8 (*CD8b1* and *Cd8a*) recognizes the virus-specific peptides presented by class I molecules.

The alteration of 5 genes (*Ctss*,* H2-Dma*,* Lgmn*,* H2-Eb1, *and *Cd4*) in MHC-II-associated pathway by the medium-dose SFXF treatment could potentially prevent virus attachment against viral infection, before virus could replicate and cause infection in host cells. Nayak et al. suggested that the characteristics of the CD4 T cell repertoire to any given pathogen, such as influenza, may be highly dependent on the array of MHC class II molecules in a given individual [13]. In our study, major histocompatibility complex II (MHC-II) genes (*H2-Oa*,* H2-Dma*,* H2-Eb1,* and *H2-Ab1*) were significantly upregulated in response to H1N1 infection. Lgmn (*AEP*) has a pivotal role to degrade the endocytosed antigens in the endosomal/lysosomal degradation system. And AEP −/− mice are unable to generate a strong anti-influenza A virus (IAV) response, as TLR7 requires a proteolytic cleavage by AEP to generate a C-terminal fragment competent for signaling [8]. Thus, SFXF may offer new therapeutic potential through AEP activity for targeting TLR7-dependent inflammatory diseases. Class II molecules are synthesized in the endoplasmic reticulum (ER) and bind the invariant chain (*Cd74*). Major histocompatibility complex class II-associated invariant chain is transported to the trans-Golgi, where cellular proteinases including cathepsins B, S (*Ctsb *and* Ctss*) cleave the invariant chain to produce a shorter, residual peptide called corticotrophin-like intermediate lobe peptide (CLIP) (*Cd74*). Then removal of CLIP by HLA-DM (*H2-Dma*) allows endocytically processed peptides to bind and stabilize the MHC proteins. The peptide-MHC class II is transported to the cell surface and recognized by CD4 T cells (*CD4*).

Fourteen genes (including *Ctla4*,* Cd247*,* Ptprc*,* Rasgrp1*,* Mapk1*,* Map3k8*,* Nfkbie*,* Nfkbib*,* Nfkbia*,* Zap70*,* Pik3r5*,* Pik3cg*,* Map2k1, *and* Pik3cd*) were downregulated by medium-dose SFXF treatment. Recognition of peptide-MHC complex by the TCR is the first signal for activation of T cell. The second signal is closely associated with CD28/CTLA-4 costimulatory pathway. Interestingly, we found that CTLA4 (*Ctla4*) expression from lung tissue of H1N1 increased 1.59-fold, but no significant difference was detected for CD28 expression. Our data was consistent with some previous studies as below. First, Ayukawa and others found that the percentages of intracellular CTLA-4-positive CD4 T cells in the patients with influenza virus infection were significantly higher than those in the healthy (*P* < 0.01) [14]. Second, Bour-Jordan and colleagues suggested that CTLA-4 had a critical role in stabilization by increasing the strength of signaling through the T cell receptor [15]. Third, Schneider's finding showed that CTLA-4 increased T cell motility and overrided the T cell receptor (TCR) induced stop signal required for stable conjugate formation between T cells and antigen-presenting cells [16]. For cytoplasmic signaling, the TCR-induced increase in tyrosine phosphorylation of the TCR zeta-chains (*CD247*) via CD45 (*Ptprc*) can recruit and activate the protein-tyrosine kinase ZAP-70 (*Zap70*), which mediates the downstream signaling cascades. Ras (*Rasgrp1*)/MEK (*Map2k1*)/ERK (*Mapk1*)/MAPK pathway, PI3 K (*Pik3r5*,* Pik3cg *and* Pik3cd*)/Akt signaling pathway, and IKK (*Nfkbie*,* Nfkbib *and* Nfkbia*)/NF-*κ*B signaling pathway then induced the expression of genes involved in the signal transduction of T cell activation in this study. In addition, in IKK/NF-*κ*B signaling pathway, it is recently reported that NS1 is also able to interact with IKK*β* and also impairs I*κ*B*α* phosphorylation and consequent translocation of NF-*κ*B dimers to the nucleus [17]. TLR engagement can control IKK activation for type I IFNs induction [18]. I*κ*B*β* acts through p65 : c-Rel dimers to maintain prolonged expression of TNF*α* [19]. IkB*ε* itself is upregulated at the mRNA level by TNF, whose overexpression is a feature of inflammatory disease [20]. This observation in our study, together with previous studies, may represent a mechanism of contributing to the activation of type I IFNs and TNF-*α* during infection and inflammatory response.

Then these signalings translocate to the nucleus and make effector T cells activate and proliferate. CTLs maturated from CD8 precursor T-cell can kill target cells by releasing perforins (*Prf1*) and granzymes (*Gzmb*). Meanwhile, CTLs induce apoptosis by triggering the Fas death receptor (*Fas*) on the surface of the target cells and binding to the Fas ligand (*Fasl*). In our study, we indicated that the notable genes in these significantly changed networks were* Gzmb* and* Tnf, *exhibiting 4.79- and 5.03-fold decreased expression exposed to the treatment of the medium-dose SFXF, respectively. As SFXF might have a role in lung tissue repair, it might protect the lung from host or virus-mediated damage. Cell deaths in these two mechanisms are within 4–6 hours. CTLs rely on the binding of TNF-*α* (*Tnf*) and its receptor to induce apoptosis in 18 hours.

A significant role for CD4 T cell in cross-protection may be supporting and enhancing CTL responses and memory cells, as well as providing help for B-cell responses via cytokine secretion [21, 22]. CD4 T cells can be divided into two different subsets, designated as Th1 and Th2 cells, which are based on the distinct lymphokine expression. IL-4 produced by Th2 cells is required for B cell proliferation and activates enzymes and molecules correlated with CD8+ cytotoxic T cells. IFN-*γ* made by Th1 cells is performed for clonal expansion of the antigen-specific T cells. So mRNA expression of either IFN-*γ* or IL-4 is evaluated in terms of Th1 and Th2 differentiation from naïve cells. The balance between Th1 and Th2 cytokine is required for the protection against influenza virus [23]. Recent reports indicated that influenza virus A infection led to Th2-biased immunity by enzyme-linked immunosorbent assay (ELISA) [24]. In our study, influenza virus infection caused Th2 polarization, too. Compared with normal control group, the expressions of IFN-*γ* mRNA and protein were significantly downregulated in virus control group, while those of IL-4 were markedly upregulated (all *P* < 0.05). The medium-dose SFXF may induce a decrease in the Th2 products with the Th1/Th2 balance and a shift from Th2 to Th1 response. After treatment of the medium-dose SFXF, all cytokines were turned to baseline levels. There was no statistical difference from the medium-dose SFXF and Oseltamivir.

Before this study, based on the guidelines issued by the Chinese Ministry of Health, the data of pyretic patients in fever clinic of Chinese-Japanese Friendship Hospital were collected by rapid influenza diagnostic tests (RIDT) from February to April, 2012. Clinical trials of this herbal granule had demonstrated its efficacy on reducing the duration of fever in patients with influenza A (H3N2) virus infection. Efficacy of a Chinese medicine granule, Shu-Feng-Xuan-Fei, had been demonstrated in reducing the duration of fever among patients with influenza A (H3N2) virus [4]. In our previous experiments *in vivo*, we treated mice with SFXF and Oseltamivir therapy for 4 consecutive days following infection with H1N1. SFXF had also been found efficacious in reducing lung index and pathological lesion (especially for inflammation severity). In three groups of SFXF from high-dose to low-dose, inhibition ratios of pulmonary-index were 15.66%, 33.31%, and 29.76%, respectively. Meanwhile, with histopathologic analyses of lung tissue of mice in virus control group, alveolar walls were diminished and infiltrated by a large number of lymphocytes, monocytes, and neutrophils. The medium-dose SFXF, as well as the Oseltamivir group, had little infiltration of inflammatory cells and tended to have mostly recovered from the primary influenza infection [25]. Moreover, we focused on innate immune response and screened for genes associated with natural killer (NK) cell mediated cytotoxicity in pneumonia mice infected with influenza virus and regulation of SFXF granules in variant doses. NK cells were activated without MHC molecules restricted early in the viral infection and later were taken over by CTLs. Apart from gene coexpression network in the high-dose and the low-dose SFXF, the medium-dose SFXF expressed extra differential genes which the high and the low did not [26]. After treatment of the medium-dose SFXF, main variants had turned to baseline levels, which showed no statistical difference from Oseltamivir. Together with them, the regulation of SFXF granules in medium-dose was the best choice for antivirus treatment in all doses of SFXF. Large amount of Chinese traditional in the high-dose SFXF medicine may delay the pharmaceutic absorption or increase toxicosis in adverse effect. Insufficient curative effect had made the low-dose SFXF undesirable to defend against viral infection.

The major ingredients of SFXF granules are based upon classical Yin-Qiao-San formula, which had been reported that could reduce time to fever resolution in patients with H1N1 influenza virus infection. Following the guidance of monarch, minister, assistant, and guide principle in basic theory of tradition Chinese medicine, the constructions of prescription are divided into principal agents and secondary agents. As principal agents in SFXF granules, indigowoad root, fructus forsythiae, as well as other single Chinese herbs, contain flavonoids, volatile oil, and agglutinin that can lead to a corresponding inhibition of the release of virus particles from infected cells, prevent influenza virus-induced deaths, and regulate immune system [27–29]. Treatment with classical herb couple of flos lonicerae and fructus forsythiae preparations with an absorption enhancer can prevent MDCK damage after influenza virus propagation [30]. Scutellaria baicalensis inhibits the replication of influenza virus H3N2 at least partly by inhibiting the fusion of viral envelopes with the endosome/lysosome membrane [31]. Monomer from folium isatidis can reduce lung index, pulmonary pathology, and hemagglutination titers over the course of influenza virus [32].

In summary, influenza virus entered and replicates in the host target cell. T cells recognized viral-specific peptides presented by MHC molecules then differentiated, proliferated, and exerted cell-mediated cytotoxic function by CTLs against influenza antigens. So the genes associated with T cell mediated activation, differentiation to CD4 and CD8 T cells, and cytotoxicity in influenza virus infection were almost upregulated, but SFXF granules could down-regulate these genes. The mechanism could be through the reduction of influenza infected cells and activation of T cells. This immunomodulation effects could be realized by regulating gene expressions of T cells activation. Viral replication was found to have been prevented and the viral infection was eliminated with exposure to SFXF granules. Thus, SFXF could help to restore a balance of the host immune system, which may be critical for viral clearance in early phase of influenza virus infection. Further studies are warranted.

## 5. Conclusion

Recently, DNA microarray analysis has been widely used in detecting the characterization of complex molecular responses in host cells following infection by H1N1 virus. We obtained whole spectrum of gene expression, important bioactivities, and the potential applications of SFXF to regulate T cell-mediated immunity in the treatment of pneumonia infected with influenza virus. Taken together, these results suggested that viral replication was prevented and the viral infection was eliminated because of exposure to SFXF granules. The mechanism could be that the reduction of influenza infected cells and T cells activation were in treatment with SFXF granules. SFXF treatment may induce immunomodulation by regulating gene expressions of T cells activation. Thus, SFXF could help to restore a balance of the host immune system, which may be critical for viral clearance in early phase of influenza virus infection.

## Figures and Tables

**Table 1:** Top 10 categorized pathways based on comparison of gene expression between normal and virus control groups.

Pathway name	Gene counts	*P* value
Cytokine-cytokine receptor interaction	87	4.1*E* − 18
Chemokine signaling pathway	58	6.4*E* − 10
MAPK signaling pathway	51	1.3*E* − 2
Jak-STAT signaling pathway	48	3.4*E* − 8
Toll-like receptor signaling pathway	39	8.8*E* − 10
Cell adhesion molecules (CAMs)	32	1.9*E* − 2
Apoptosis	32	2.4*E* − 7
T-cell receptor signaling pathway	31	5.5*E* − 4
Leukocyte transendothelial migration	24	5.9*E* − 2
Antigen processing and presentation	23	5.6*E* − 3

**Table 2:** Genes associated with Toll-like receptor signaling pathway.

Symbol	GenBank accession	Gene description	log_2_ FC^a^				
			M/N	SH/M	SM/M	SL/M	Oseltamivir/M
*Tlr7 *	mMC009252	Toll-like receptor 7, TLR7	1.39	0.02	−2.02	−0.43	−0.92
*MyD88 *	PH_mM_0009196	Myeloid differentiation primary response gene 88	2.22	−1.18	−2.63	−1.36	−1.62
*Mapk8 *	PH_mM_0009123	Mitogen-activated protein kinase 8, JNK	1.82	−1.14	−1.22	−1.04	−1.24
*Mapk13 *	PH_mM_0004240	Mitogen-activated protein kinase 13, P38	1.39	−2.27	−1.89	−2.10	−1.50
*Tnf *	mMC016605	Tumor necrosis factor, TNF-*α*	5.47	−3.86	−5.03	−4.58	−4.60
*Il1b *	mMC010962	Interleukin 1 beta, IL-1*β*	3.69	−2.92	−4.27	−2.33	−2.47
*Cxcr2 *	PH_mMC_0000540	Chemokine (C-X-C motif) receptor 2, IL-8	1.93	−2.80	−3.25	−2.25	−2.22
*Ccl5 *	mMC0118383	Chemokine (C-C motif) ligand 5, RANTES	3.51	−0.93	−3.39	−1.95	−2.26
*Tgfb1 *	PH_mMC_0001301	Transforming growth factor, beta 1	1.46	−0.84	−1.25	−0.92	−1.35
*Csf2 *	mMC014357	Colony stimulating factor 2	2.41	−1.40	−2.03	−1.43	−2.19
*Il18 *	mMC021817	Interleukin 18, IL-18	2.02	−0.66	−1.10	−0.76	−1.26
*Il12a *	PH_mM_0009192	Interleukin 12a, IL-12	3.38	−3.32	−2.92	−2.91	−3.05

^a^Fold change. The intensity ratio of probe signal criteria for differential expressions was defined as a twofold change (up or down) (log_2_ FC ≥ 1 or ≤−1) in gene expression compared with virus control group and a *P* value less than 0.05 in at least one time point as a minimum requirement to select. For instance, log_2_ (M/N) ≥ 1 means that the comparison between normal control group and virus control group was scattered, indicating that a large number of genes in virus control group were up-regulated in response to H1N1 infection. log_2_ (SH/M) ≤−1, log_2_ (SM/M) ≤−1, log_2_ (SL/M) ≤−1, and log_2_ (Oseltamivir/M) ≤−1 means that down-regulation in gene expression attributable to the high-dose SFXF group treatment, medium-dose group SFXF treatment, low-dose SFXF group treatment, and Oseltamivir group treatment was done by comparing genes in virus control group, respectively (*P* value data not shown).

**Table 3:** Genes associated with antigen processing presentation.

Symbol	GenBank accession	Gene description	log_2_ FC^a^				
			M/N	SH/M	SM/M	SL/M	Oseltamivir/M
*Tap1 *	PH_mM_0000666	Transporter 1, ATP-binding cassette, subfamily B (MDR/TAP)	2.24	−0.30	−2.83	−0.41	−1.00
*Tap2 *	mMC017161	Transporter 2, ATP-binding cassette, subfamily B (MDR/TAP)	2.29	−0.02	−2.27	−0.20	−1.05
*Cd74 *	PH_mM_0016294	CD74 antigen (invariant polypeptide of major histocompatibility complex, class II antigen-associated), also as CLIP	1.74	0.47	0.60	0.13	−0.75
*Ctsb *	PH_mM_0013880	Cathepsin B	1.47	−0.27	−0.95	−0.24	−0.54
*Ctss *	PH_mM_0009536	Cathepsin S	1.99	0.29	−1.39	−0.28	−0.90
*Hspa5 *	mMC021274	Heat shock protein 5	1.27	0.78	0.22	0.29	−0.16
*Hspa1a *	PH_mM_0000552	Heat shock protein 1A	2.05	−0.17	−2.39	−0.67	−0.75
*Hspa1b *	PH_mM_0001288	Heat shock protein 1B	1.53	0.63	−1.08	−0.47	−0.18
*H2-M2 *	PH_mM_0002687	Histocompatibility 2, M region locus 2	2.27	−0.64	−2.16	−1.89	−1.47
*H2-M3 *	PH_mM_0009138	Histocompatibility 2, M region locus 3	2.37	−0.24	−2.10	−0.55	−1.39
*H2-Oa *	PH_mM_0000005	Histocompatibility 2, O region alpha locus	1.15	−0.38	−0.44	−0.55	−0.67
*H2-Q10 *	PH_mM_0005188	Histocompatibility 2, Q region locus 10	5.62	−2.04	−2.72	−3.84	−4.12
*H2-Dma *	PH_mM_0005361	Histocompatibility 2, class II, locus DMa	1.49	−0.17	−1.11	−0.43	−1.14
*H2-Eb1 *	PH_mM_0006643	Histocompatibility 2, class II antigen E beta	1.30	−0.31	−1.87	−1.31	−1.33
*H2-Ab1 *	PH_mM_0006906	Histocompatibility 2, class II antigen A, beta 1	1.17	0.26	−0.05	0.33	−0.10
*Lgmn *	mMC007543	Legumain	2.27	0.08	−1.10	−0.24	−0.83
*Lta *	mMC008431	Lymphotoxin A, TNF-*β*	1.34	−0.14	−1.17	−0.18	−1.19
*Psme1 *	PH_mM_0004486	Proteasome (prosome, macropain) 28 subunit, alpha	1.00	−0.40	−1.16	−0.53	−0.50
*H2-T24 *	PH_mM_0007562	Histocompatibility 2, T region locus 24	2.82	−0.15	−2.19	0.21	−1.72
*Psme2 *	PH_mM_0012374	Proteasome (prosome, macropain) 28 subunit, beta	1.99	−1.00	−1.55	−1.58	−1.00
*Cd8a *	PH_mM_0015300	CD8 antigen, alpha chain	2.27	1.20	−1.65	−0.42	−1.05
*Cd8b1 *	mMC017400	CD8 antigen, beta chain 1	2.49	1.31	−1.18	−1.58	−0.73
*Cd4 *	mMC002333	CD4 antigen	1.05	−0.32	−1.11	−0.92	−1.00

^a^Fold change. The intensity ratio of probe signal criteria for differential expressions was defined as a twofold change (up or down) (log_2_ FC ≥ 1 or ≤−1) in gene expression compared with virus control group and a *P* value less than 0.05 in at least one time point as a minimum requirement to select. For instance, log_2_ (M/N) ≥ 1 means that the comparison between normal control group and virus control group was scattered, indicating that a large number of genes in virus control group were up-regulated in response to H1N1 infection. log_2_ (SH/M) ≤−1, log_2_ (SM/M) ≤ −1, log_2_ (SL/M) ≤ −1, and log_2_ (Oseltamivir/M) ≤ −1 means that down-regulation in gene expression attributable to the high-dose SFXF group treatment, medium-dose group SFXF treatment, low-dose SFXF group treatment, and Oseltamivir group treatment was done by comparing genes in virus control group, respectively (*P* value data not shown).

**Table 4:** Genes associated with T-cell receptor signaling pathway.

Symbol	GenBank accession	Gene description	log_2_ FC^a^				
			M/N	SH/M	SM/M	SL/M	Oseltamivir/M
*Cd3g *	PH_mM_0008250	CD3 antigen, gamma polypeptide, CD3*γ*	1.26	1.43	−0.50	0.17	−0.49
*Cd247 *	PH_mM_0012514	CD247 antigen, CD3*ζ*	1.56	0.38	−1.59	−0.65	−1.10
*Ptprc *	PH_mM_0012802	Protein tyrosine phosphatase, receptor type, C. CD45	1.65	0.32	−1.41	−0.11	−0.52
*Rasgrp1 *	PH_mM_0009315	RAS guanyl releasing protein 1	2.43	−0.93	−1.94	−1.25	−1.60
*Ctla4 *	mMC018651	Cytotoxic T-lymphocyte-associated protein 4	1.59	0.67	−1.94	−0.49	−1.01
*Ikbkb *	mMR027870	Inhibitor of kappaB kinase beta	1.33	−0.65	−0.87	−0.67	−1.19
*Mapk1 *	mMC024552	Mitogen-activated protein kinase 1, Erk	2.23	−0.90	−1.04	−0.47	−1.50
*Map3k8 *	mMC012006	Mitogen-activated protein kinase kinase kinase 8, COT	3.59	−2.86	−3.98	−2.92	−3.09
*Nfkbie *	PH_mM_0006219	Nuclear factor of kappa light polypeptide gene enhancer in B cells inhibitor, epsilon. I*κ*B*ε*	4.26	−1.84	−3.75	−2.32	−3.13
*Nfkbib *	mMC008604	Nuclear factor of kappa light polypeptide gene enhancer in B cells inhibitor, beta. I*κ*B*β*	3.34	−2.35	−2.68	−2.12	−2.88
*Nfkbia *	mMC012597	Nuclear factor of kappa light polypeptide gene enhancer in B cells inhibitor, alpha. I*κ*B*α*	1.66	−0.92	−1.95	−1.33	−1.52
*Zap70 *	mMC019091	Zeta-chain associated protein kinase, ZAP-70	1.53	0.38	−1.36	−0.58	−1.49
*Pik3r5 *	PH_mM_0003125	Phosphoinositide-3-kinase, regulatory subunit 5, p101. PI3K	3.12	−2.52	−3.90	−2.95	−2.88
*Pik3cg *	mMC005200	Phosphoinositide-3-kinase, catalytic, gamma polypeptide. PI3K	1.64	−0.75	−1.49	−1.25	−1.21
*Map2k1 *	PH_mM_0006215	Mitogen-activated protein kinase kinase 1	1.76	−0.53	−1.00	−0.81	−1.06
*Pik3cd *	PH_mM_0001034	Phosphatidylinositol 3-kinase catalytic delta polypeptide. PI3K	1.42	−0.27	−2.08	−0.74	−0.96

^a^Fold change. The intensity ratio of probe signal criteria for differential expressions was defined as a twofold change (up or down) (log_2_ FC ≥ 1 or ≤−1) in gene expression compared with virus control group and a *P* value less than 0.05 in at least one time point as a minimum requirement to select. For instance, log_2_ (M/N) ≥ 1 means that the comparison between normal control group and virus control group was scattered, indicating that a large number of genes in virus control group were up-regulated in response to H1N1 infection. log_2_ (SH/M) ≤ −1, log_2_ (SM/M) ≤ −1, log_2_ (SL/M) ≤ −1, and log_2_ (Oseltamivir/M) ≤ −1 means that down-regulation in gene expression attributable to the high-dose SFXF group treatment, medium-dose group SFXF treatment, low-dose SFXF group treatment and Oseltamivir group treatment was done by comparing genes in virus control group, respectively (*P* value data not shown).

**Table 5:** Genes associated with effector T cells activation and cytokines involved in T-cell-mediated immunity.

symbol	GenBank accession	Gene description	log_2_ FC^a^				
			M/N	SH/M	SM/M	SL/M	Oseltamivir/M
*Gzmb *	PH_mM_0001308	Granzyme B	4.36	1.17	−4.79	−0.13	−1.54
*Prf1 *	mMC017608	Perforin 1	2.43	0.31	−2.71	−0.55	−1.55
*Fas *	mMC008894	Tumor necrosis factor receptor superfamily member 6	1.70	−1.13	−1.83	−0.88	−1.70
*FasL *	mMC013655	Fas ligand	1.91	−1.49	−2.13	−0.08	−1.07
*Il4 *	PH_mM_0000958	Interleukin 4, IL-4	5.43	−1.00	−5.19	−1.33	−2.64
*Ifng *	PH_mM_0001407	Interferon-gamma, IFN-*γ*	−1.02	1.28	1.23	1.38	1.35

^a^The intensity ratio of probe signal criteria for differential expressions was defined as a twofold change (up or down) (log_2_ ratio ≥ 1 or ≤−1) in gene expression compared with virus control group and a *P* value less than 0.05 in at least one time point as a minimum requirement to select. For instance, log_2_ (M/N) ≥ 1 means that the comparison between normal control group and virus control group was scattered, indicating that a large number of genes in virus control group were up-regulated in response to H1N1 infection. log_2_ (SH/M) ≤ −1, log_2_ (SM/M) ≤ −1, log_2_ (SL/M) ≤ −1, and log_2_ (Oseltamivir/M) ≤ −1 means that down-regulation in gene expression attributable to the high-dose SFXF group treatment, medium-dose SFXF group treatment, low-dose SFXF group treatment, and Oseltamivir group treatment was done by comparing genes in virus control group, respectively (*P* value data not shown).
